# Lipid-bound apolipoproteins in tyrosyl radical-oxidized HDL stabilize ABCA1 like lipid-free apolipoprotein A-I

**DOI:** 10.1186/1471-2091-13-1

**Published:** 2012-01-16

**Authors:** Mohammad A Hossain, Sereyrath Ngeth, Teddy Chan, Michael N Oda, Gordon A Francis

**Affiliations:** 1 Department of Medicine, UBC James Hogg Research Centre, Heart and Lung Institute, St. Paul's Hospital, Vancouver, British Columbia, Canada V6Z 1Y6; 2 Children's Hospital Oakland Research Institute, Oakland, CA, USA 94609

## Abstract

**Background:**

ATP-binding cassette transporter A1 (ABCA1) mediates the lipidation of exchangeable apolipoproteins, the rate-limiting step in the formation of high density lipoproteins (HDL). We previously demonstrated that HDL oxidized ex vivo by peroxidase-generated tyrosyl radical (tyrosylated HDL, tyrHDL) increases the availability of cellular cholesterol for efflux and reduces the development of atherosclerosis when administered to apolipoprotein E-deficient mice as compared to treatment with control HDL.

**Results:**

In the current study we determined that tyrHDL requires functional ABCA1 for this enhanced activity. Like lipid-free apolipoprotein A-I (apoA-I), tyrHDL increases total and cell surface ABCA1, inhibits calpain-dependent and -independent proteolysis of ABCA1, and can be bound by cell surface ABCA1 in human skin fibroblasts. Additionally, tyrHDL apoproteins are susceptible to digestion by enteropeptidase like lipid-free apoA-I, but unlike lipid-bound apoA-I on HDL, which is resistant to proteolysis.

**Conclusions:**

These results provide the first evidence that lipid-bound apolipoproteins on the surface of spherical HDL particles can behave like lipid-free apoA-I to increase ABCA1 protein levels and activity.

## Background

High density lipoprotein cholesterol (HDL-C) levels in human plasma correlate generally with protection against coronary heart disease, an effect believed to be due to multiple protective actions of HDL including stimulating the removal of excess cholesterol from cells and reducing inflammation in the artery wall [1]. The initial formation of HDL particles requires the membrane lipid transporter ATP-binding cassette transporter A1 (ABCA1) to mediate delivery of cellular lipids to HDL apolipoproteins. This reduces excess cholesterol stores in cells including arterial wall macrophages [2]. In addition to receiving cellular lipids, lipid-free apolipoprotein A-I (apoA-I) binds to and inhibits the degradation of ABCA1, further enhancing the formation of HDL particles [3,4]. Lipidated apoA-I on the surface of discoidal or spherical HDL particles, however, has a reduced affinity for ABCA1 and does not inhibit ABCA1 degradation [3,5]. These findings suggest structural motifs present in lipid-free but not lipid-bound apoA-I participate in ABCA1 binding and enhancement of ABCA1 cell surface stability.

In addition to increasing ABCA1 expression transcriptionally, therapies that increase ABCA1 cell surface stability represent a potential means to increase HDL production. We previously showed that oxidation of HDL ex vivo with peroxidase-generated tyrosyl radical (tyrosylated HDL or tyrHDL) increases the ability of HDL to deplete the regulatory pool of intracellular cholesterol [6], increase cholesterol available for subsequent efflux to apoA-I and other lipid acceptors [7], and reduce the development of atherosclerosis in apoE-deficient mice [8]. Enhanced depletion of intracellular cholesterol was seen only when tyrHDL apoproteins and specifically the apoAI-apoAII heterodimer in tyrHDL were bound to the surface of spherical HDL particles, but not in their lipid-free form. This suggests a particular conformation of tyrHDL apos when lipid-bound mediates the enhanced effect [9]. In the current study we tested the hypothesis that tyrHDL has these beneficial effects by enhancing ABCA1 activity, either by increasing ABCA1 expression or cell surface stability. Our results indicate that, like lipid-free apoAI, tyrHDL has no effect on ABCA1 mRNA levels, but increases total and cell surface ABCA1, reduces calpain-dependent and -independent degradation of ABCA1, and can be crosslinked directly to ABCA1. Also like lipid-free apoA-I but not HDL apos, apos on the surface of tyrHDL are more susceptible to enteropeptidase digestion. These results suggest that tyrosyl radical oxidation induces changes in apolipoprotein conformation on the surface of spherical HDL that allows lipid-bound tyrHDL apos to interact with ABCA1 like lipid-free apoA-I.

## Methods

### Materials

Cholesterol, L-tyrosine, horseradish peroxidase, hydrogen peroxide (30%, ACS grade), dietheylenetriaminepentaacetic acid (DTPA; free acid form), essentially fatty acid-free bovine serum albumin (BSA), N-acetyl-Leu-Leu-norleucinal (ALLN), LXR agonist T0901317 and fetal bovine serum were purchased from Sigma. [^14^C]Oleate (55 mCi/mmol) was from GE Healthcare. Dulbecco's modified Eagle's medium (DMEM) was purchased from Hyclone. PE-SIL G plastic backed flexible plates used for thin-layer chromatography analysis were from Whatman. Nitrocellulose membranes, sodium dodecyl sulfate polyacrylamide gel electrophoresis (SDS-PAGE) reagents, pre-stained protein molecular mass markers and Chelex 100 resin were from Bio-Rad. μ-Calpain was from Calbiochem, and Trizol from Invitrogen. Cross-linking agent dithiobis(succinimidyl) propionate (DSP) was from Pierce.

### Preparation of HDL, apolipoprotein A-I, and tyrosylated HDL

HDL_3 _was isolated from pooled plasma from healthy fasting donors by density gradient ultracentrifugation [10]. Apo A-I was purified from human plasma using Q-Sepharose Fast Flow chromatography as previously described [11]. Tyrosylation of HDL was carried out at 37°C for 24 h in 66 mM potassium phosphate buffer, pH 8.0, which had been passed over Chelex 100 resin to remove transition metal ions. The reaction mixture contained a final concentration of 1 mg/mL HDL protein, 100 *μ*M diethylenetriaminepentaacetic acid (to inhibit metal ion-catalyzed oxidation), 100 nM horseradish peroxidase (250 units/mg), 100 *μ*M H2O2, and 100 μM L-tyrosine. The reaction mixture was subjected to size exclusion chromatography as previously described [7] prior to use in cell culture experiments.

### Cell culture

Human skin fibroblasts were cultured in DMEM supplemented with 10% FBS containing 50 units/ml penicillin-streptomycin solution (Invitrogen) and grown in humidified 95% air and 5% CO_2 _at 37°C. Confluent cells were rinsed twice with phosphate-buffered saline (PBS) containing 1 mg/ml BSA (PBS-BSA) and incubated for 24 h in DMEM containing 2 mg/ml BSA with 30 μg/ml non-lipoprotein cholesterol. To allow equilibration of added cholesterol, cells were rinsed twice with PBS-BSA and incubated for an additional 24 h in DMEM containing 1 mg/ml BSA (DMEM-BSA).

### Cholesterol esterification assay

Cholesterol-loaded cells were incubated for 16 h in DMEM-BSA and the indicated additions, washed once with PBS, and incubated for 1 h at 37°C with DMEM containing 9 mM [^14^C] oleate bound to 3 mM BSA [12]. Cells were chilled on ice and rinsed twice with ice-cold PBS-BSA, twice with PBS, and stored at -20°C until extraction. Cellular lipids were extracted with hexane:isopropanol (3:2, v/v). Sterol species were separated by thin layer chromatography (TLC) on PE SIL G plastic-backed plates (Whatman) developed in hexane/diethyl ether/acetic acid (130:40:1.5 v/v/v). Lipid spots were identified by staining with I_2 _vapor and co-migration with standard. After allowing I_2 _stain to evaporate, cholesteryl ester spots were taken for determination of radioactivity by liquid scintillation counting. Cell protein was extracted by incubation of cells with 0.5 mL of 0.1 N NaOH for 1 h on a rotary shaker. Total cell protein was determined by the Lowry assay [13] using albumin as a standard.

### Reverse transcriptase-PCR analysis of ABCA1 mRNA

Total RNA was isolated from cells using Trizol, following manufacturer's protocols. Single-strand cDNA was synthesized by a SuperScript pre-amplification system (Invitrogen) from 2 μg of the total RNA. ABCA1 DNA amplification was performed by initial denaturation at 95°C for 3 mins. Thereafter, denaturing was at 95°C for 20 seconds, annealing at 58°C for 20 seconds, and extension at 72°C for 40 seconds for at total of 40 cycles (10). SYBR Green (Quanta Biosciences) was used to detect PCR products in real-time using a Realplex^2 ^Mastercycler thermocycler (Eppendorf). The human housekeeping gene cyclophilin cDNA was amplified using the same conditions. ABCA1 mRNA levels were calculated using the comparative C_T _method relative to cyclophilin. The following primers were used: human ABCA1, 5'-GAC ATC CTG AAG CCA ATC CTG (forward), 5'-CCT TGT GGC TGG AGT GTC AGG T (reverse); human cyclophilin, 5'- ACC CAA AGG GAA CTG CAG CGA GAG C (forward), 5'-CCG CGT CTC CTT TGA GCT GTT TGC AG (reverse).

### Gel electrophoresis and immunoblotting

Cells were scraped into N-dodecyl-β-D-maltoside--containing lysis buffer (20 mM tris, 5 mM EDTA, 5 mM EGTA, 0.5% N-dodecyl-β-D-maltoside, pH 7.5) with complete protease inhibitor (Roche Molecular Biochemicals) and stored at -80°C. Cells were homogenized by sonication, centrifuged to remove the cellular debris, and the lysate collected for ABCA1 protein detection. The cell lysate was run on a 5-15% gradient SDS-PAGE gel under reducing conditions and transferred to nitrocellulose membrane for 16 h at 4°C. Immunoblotting was performed using a polyclonal rabbit anti-human ABCA1 antibody from Novus Biologicals (#NB400-105, 1:1000 dilution) and a goat anti-rabbit IgG horseradish perodixase-conjugated secondary antibody from Sigma (#A-0545, 1:10,000 dilution). Immunoblots were re-probed with rabbit polyclonal anti-actin (Abcam, #AB8227-50, 1:2000) as loading control. SDS-PAGE was also performed for the detection of apoA-I and apoA-II using 12% polyacrylamide gels under reducing conditions followed by staining with 0.25% Coomassie Brilliant Blue. Immunoblot analysis of apoA-I, HDL and tyrHDL were performed using rabbit anti-human apoA-I polyclonal antibody (Calbiochem #178422, 1:10,000) and goat anti-human apoA-II monoclonal antibody (Calbiochem, #178464, 1:20,000).

### Calpain and ALLN studies

Sensitivity of ABCA1 to digestion by exogenous calpain following incubation with apoA-I, HDL, or tyrHDL was performed using digitonin permeabilization and treatment with 0.5 μM μcalpain for 20 min as previously described [4]. To assess cellular levels of ABCA1 following inhibition of endogenous calpain-dependent degradation by ALLN, cells were incubated for 16 h with the indicated conditions plus 50 μM ALLN as previously described [3]. Cell lysates were then analyzed by ABCA1 immunoblotting as described above.

### Biotinylation of cell-surface proteins

Proteins on the surface of cells treated with the indicated additions were biotinylated, harvested, and purified on streptavidin resin using a Pierce Cell Surface Protein Isolation Kit (Pierce) following manufacturer's protocols [14]. Briefly, after surface biotinylation and quenching, cells were lysed and debris was removed by centrifugation at 10,000 × g for 10 min. Biotinylated proteins in the supernatant (500 μl) were bound to a streptavidin column and eluted in 250 μl of SDS sample buffer. The total and cell surface fraction were treated with 50 mM dithiothreitol to remove biotin, resolved by SDS-PAGE, and immunoblotted using antibodies against ABCA1, β-actin (Abcam), and ERK (New England Biolabs).

### Crosslinking of apoA-I, HDL or tyrHDL to ABCA1

Chemical cross-linking was performed as described by Wang et al. [15] with minor modifications as follows. Cells were grown to confluence in 100 mm culture dishes, cholesterol loaded with non-lipoprotein cholesterol, equilibrated, and then treated with 5 μM LXR Agonist (T0901317) in DMEM-BSA for 24 h to up-regulate ABCA1 expression. The cells were washed twice with PBS and then treated with DMEM/BSA or the same media containing 10 μg/mL apoA-I, HDL, or tyrHDL in DMEM-BSA for 2 h at 37°C. Cells were then placed on ice for 15 min and washed three times with PBS. Cross linking agent DSP was dissolved immediately before use in dimethyl sulfoxide (Sigma) and diluted to 250 μM with PBS. 10 ml of DSP solution was added to the cells for 1 h at room temperature, the medium removed, and dishes were washed twice with ice-cold PBS. Cells were lysed at 4°C in buffer containing 20 mM Tris, 0.5 mM EDTA, 0.5 mM EGTA, 1% Triton X-100 (BDH Chemicals, pH 7.5), with complete protease inhibitor (Roche). by aspiration with a fine-tipped needle, and the mixture was left to rotate at 4°C for 30 min. Cell lysates were centrifuged at 1,500 rpm for 10 min at 4°C to remove cellular debris The supernatant was collected and incubated with loading buffer in the absence or presence of β-mercaptoethanol before loading onto 4-20% gradient SDS-PAGE gels for electrophoresis followed by electrotransfer to a nitrocellulose sheet for immunoblot analysis. ApoA-I cross-linked to ABCA1 was detected by blotting the membranes with rabbit polyclonal anti-human apoA-I antibody and goat anti-rabbit IgG horseradish peroxidase-conjugated secondary antibody as noted above. To detect ABCA1, the membrane was stripped and reprobed with rabbit polyclonal antibody to ABCA1 and goat anti-rabbit IgG horseradish peroxidase-conjugated secondary antibody as noted above.

### Enteropeptidase digestion

Lipid-free apoA-I, HDL and tyrHDL were incubated with 0.13 U enteropeptidase (Calbiochem) per g of protein for 6 h at 37°C as previously described for apoA-I [16]. Cleaved products were run on 12% SDS-PAGE under reducing conditions and stained with 0.25% Coomassie brilliant blue. Immunoblot analysis of apoA-I and apoA-II were performed using rabbit anti-human apoA-I polyclonal antibodies and goat anti-human apoA-II monoclonal antibodies.

### Statistics

**Figure 1:**
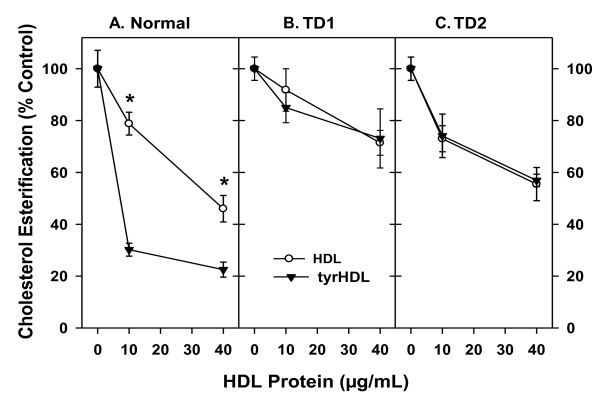
**tyrHDL requires ABCA1 to enhance depletion of ACAT-accessible cholesterol**. Human skin fibroblasts from a normal donor and 2 unrelated Tangier Disease (TD) subjects were grown to confluence, loaded with non-lipoprotein cholesterol for 24 h, equilibrated for 24 h in DMEM containing 1 mg/ml BSA, and then incubated in the same medium plus the indicated concentration of HDL or tyrHDL for 16 h. To measure residual ACAT-accessible cholesterol, cells were then incubated with [^14^C]oleate for 1 h, and cholesteryl [^14^C]esters present in cellular lipid extracts were determined. Results are expressed as percentage of cholesterol esterification in cells untreated with HDL or tyrHDL. Avg ± SD of quadruplicates, representative of 3 experiments. *, p < 0.01 when compared with tyrHDL-treated cells.

**Figure 2:**
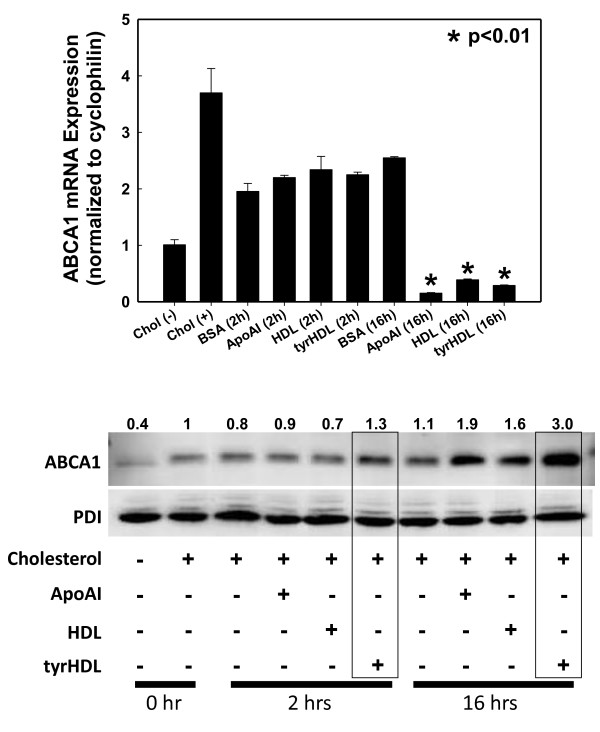
**tyrHDL increases ABCA1 protein but not mRNA levels**. Fibroblasts grown to confluence, cholesterol loaded, and equilibrated as in Figure 1 were treated with medium containing 1 mg/ml BSA alone or plus 10 μg/ml apoA-I, HDL or tyrHDL for 2 or 16 h. (A) mRNA was collected using Trizol™ extraction at the indicated time and qPCR was performed with cyclophilin used as the internal control. Data represent Avg ± SD for triplicates of each sample and are representative of three experiments with similar results. *, p < 0.01 when compared with BSA-treated cells at 16 h. (B) Cells were collected in N-dodecyl-β-D-maltoside lysis buffer with proteinase inhibitors, and ABCA1 protein was determined following separation of proteins on 5-15% gradient gels and immunoblotting. Values at the top of lanes represent the ratio of densitometer readings of ABCA1 protein normalized for protein disulfide isomerase (PDI) loading control, with this ratio in cholesterol-loaded cells at 0 h set as 1. The data are representative of three experiments with similar results. Chol- indicates cells not loaded with cholesterol loading prior to analyses.

Results for Figures 1 and 2A were analyzed using GraphPad Prism version 5.0 and are presented as the mean ± S.D. Significant differences between experimental groups were determined using the Student's *t *test.

## Results

### tyrHDL requires ABCA1 to deplete cholesterol available for esterification

Lipid-free apoA-I depletes the regulatory pool of cholesterol available for esterification by acyl-CoA:cholesterol acyltransferase (ACAT) much more readily than HDL [17], due to mobilization of this cholesterol pool by ABCA1 and delivery to apoA-I [2]. tyrHDL mobilizes ACAT-accessible cholesterol much more readily than HDL, an effect that was not found to be due to direct inhibition of ACAT or stimulation of cholesteryl ester hydrolysis [6,7]. To investigate the role of functional ABCA1 in this activity, HDL and tyrHDL were incubated with cholesterol-loaded fibroblasts from control and two unrelated patients with Tangier Disease, and the subsequent production of cholesteryl[^14^C]oleate quantified. As reported previously [6], tyrHDL showed a markedly increased ability to deplete ACAT-accessible cholesterol from normal fibroblasts in comparison to control HDL (Figure 1A). tyrHDL did not have an enhanced ability to mobilize ACAT-accessible cholesterol compared to HDL from Tangier Disease fibroblasts, and depletion of this pool was reduced to similar levels by both forms of HDL in comparison to normal cells (Figure 1B and 1C). This suggests tyrHDL requires ABCA1 for its enhanced cholesterol-mobilizing effect.

### tyrHDL increases ABCA1 protein but not mRNA levels

To test whether tyrHDL has an effect on ABCA1 transcription, cholesterol-loaded fibroblasts were incubated with either fatty acid-free bovine serum albumin (BSA) alone or with the addition of 10 μg/mL apoA-I, HDL, or tyrHDL for 2 h or 16 h prior to determination of ABCA1 mRNA level by real-time PCR. At 2 h, ABCA1 mRNA level was reduced similarly by all treatments when compared to cholesterol-loaded cells at 0 h (Figure 2A). Following a 16 h incubation, ABCA1 mRNA remained elevated in cells treated with BSA alone, but was reduced similarly to low levels by apoAI, HDL, and tyrHDL, consistent with depletion of cellular cholesterol regulating ABCA1 expression by all of these treatments. These results are consistent with previous results showing no effect of apoA-I on ABCA1 mRNA level [3], and suggest no differential effect of tyrHDL on ABCA1 transcription when compared to apoA-I or HDL.

ApoA-I has previously been shown to increase ABCA1 protein levels in cultured cells and in the liver of apoA-I-injected mice [3,4]. Consistent with these findings, total cell homogenates obtained from cholesterol-loaded fibroblasts incubated for 16 h with 10 μg/mL apoA-I exhibited higher levels of ABCA1 protein than cells treated with albumin alone or similar levels of HDL (Figure 2B). Cells treated with 10 μg/mL tyrHDL showed increased levels of ABCA1 protein when compared to apoA-I, HDL or BSA treatments at both 2 and 16 h. These results suggest tyrHDL has an enhanced ability to stabilize ABCA1 protein post-translationally when compared to apoA-I and control HDL.

### tyrHDL protects ABCA1 from calpain-dependent and -independent proteolysis

**Figure 3:**
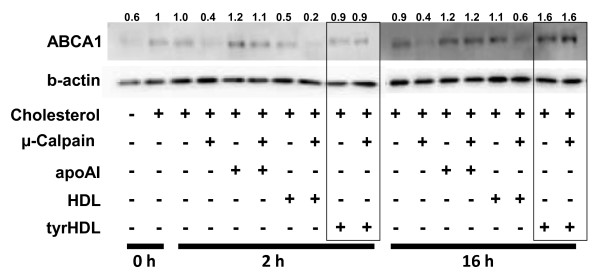
**tyrHDL protects ABCA1 from calpain-mediated degradation**. Cholesterol-loaded fibroblasts pretreated with BSA plus 10 μg/ml apoA-I, HDL, or tyrHDL for 2 h or 16 h were then incubated with or without 0.5 μM μ-calpain following digitonin permeabilization. ABCA1 protein was analyzed by 5-15% SDS-PAGE gradient gels followed by immunoblotting. Results of duplicate incubations with apoA-I, HDL, and tyrHDL are shown. The data are representative of three experiments with similar results.

Calpain-dependent proteolysis of ABCA1 is mediated through a proline, glutamic acid, serine, and threonine-rich (PEST) sequence [4], and is inhibited by lipid-free apoA-I but not HDL [3,4]. To determine whether tyrHDL inhibits calpain-dependent proteolysis of ABCA1, cholesterol-loaded fibroblasts were incubated with BSA alone or plus apoA-I, HDL or tyrHDL for 2 or 16 h, followed by treatment with 0.5 μM μ-calpain for 20 min [4]. ABCA1 protein degradation by μ-calpain was observed in BSA and HDL treated fibroblasts at both time points, with a 50-60% reduction in ABCA1 protein when compared to the absence of μ-calpain (Figure 3). No appreciable degradation of ABCA1 by calpain was observed in apoA-I- or tyrHDL-treated cells at either time point (Figure 3).

**Figure 4:**
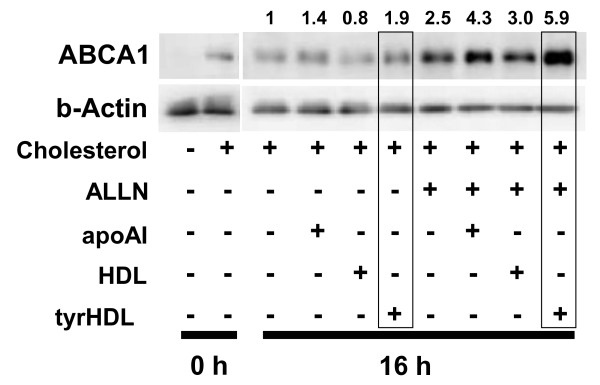
**tyrHDL increases cellular ABCA1 in the presence of ALLN**. Cholesterol-loaded fibroblasts were incubated with BSA and 10 μg/ml apoA-I, HDL, or tyrHDL with or without protease inhibitor ALLN (50 μM) to inhibit endogenous calpain for 16 h. ABCA1 was determined by immunoblotting as in Figure 3. The data are representative of three experiments with similar results.

Lipid-free apoA-I also increases ABCA1 protein levels by interfering with calpain-independent proteolysis of ABCA1 [3]. Increased ABCA1 protein seen in the presence of the calpain inhibitor ALLN and BSA alone at 16 h was increased further by apoA-I, and most markedly by incubation with tyrHDL (Figure 4). These results indicate tyrHDL inhibits calpain-dependent and -independent proteolysis of ABCA1 to a level similar or greater than lipid-free apoA-I.

### tyrHDL increases cell surface ABCA1 like lipid-free apoA-I

**Figure 5:**
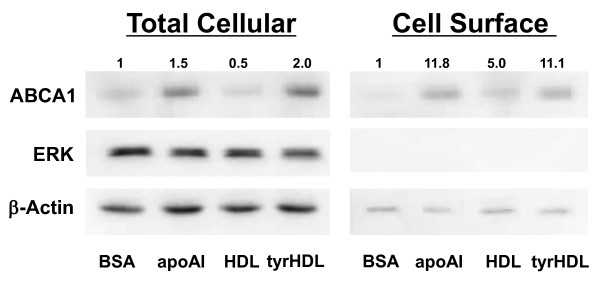
**tyrHDL increases cell surface ABCA1**. Cholesterol-loaded fibroblasts were incubated with BSA alone or with 10 μg/ml apoA-I, HDL or tyrHDL for 16 h. Total cellular ABCA1 protein was determined from whole cell lysates. Cell surface ABCA1 was determined following biotinylation and precipitation of biotinylated cell surface proteins. Total cellular and cell surface samples were run on the same 5-15% SDS-Page gradient gel followed by immunoblotting. Data are representative of three separate experiments with similar results.

ApoA-I-dependent reduction of ABCA1 proteolysis results in increased cell surface as well as total cellular ABCA1 [18]. To determine the effect of tyrHDL on cell surface ABCA1, cells incubated with either BSA alone or with addition of 10 μg/mL apoA-I, HDL, or tyrHDL for 16 h were then biotinylated to label cell surface proteins. Total and biotinylated cell surface ABCA1 were isolated and probed for ABCA1, ERK (a cytoplasmic protein), and β-actin proteins by immunoblot. Increased cell surface ABCA1 was seen with both apoA-I and tyrHDL treatment, paralleling the increase in total cellular ABCA1, and to a greater extent than seen with HDL treatment (Figure 5).

### tyrHDL forms crosslinks with ABCA1 similar to lipid-free apoA-I

**Figure 6:**
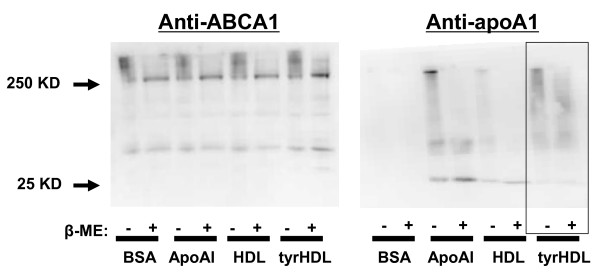
**Crosslinking of tyrHDL to ABCA1**. Cholesterol-loaded fibroblasts were equilibrated in the presence of 5 μM T0901317 for 24 h to upregulate ABCA1 expression. The cells were then treated with BSA alone or plus 10 μg/ml apoA-I, HDL and tyrHDL for 2 h, washed, and then incubated with 250 mM of DSP for 1 h. Whole cell lysates were isolated, run on a 5-15% SDS-PAGE gradient gel and probed for the presence of ABCA1 and apoA-I by immunoblot. 5% beta mercaptoethanol (β-ME) was used as a reducing agent to cleave crosslinks formed by DSP. Results are representative of three separate experiments with similar results.

Several studies using the chemical cross-linker dithiobis(succinimidyl) propionate (DSP) have suggested apoA-I can directly bind to ABCA1, whereas HDL does not [15,19,20]. These findings indicate conformational features present in lipid-free but not lipid-bound apoA-I allow the lipid-free form to bind to ABCA1. To determine whether tyrHDL might also bind to ABCA1 like lipid-free apoA-I, cholesterol-loaded fibroblasts were treated with LXR agonist T0901317 to upregulate cell surface ABCA1 protein expression, and then incubated with either BSA alone or with addition of apoA-I, HDL, or tyrHDL for 2 h, followed by incubation with DSP for an additional hour, and separation of whole cell homogenates for immunoblotting as described [15]. ApoA-I colocalized with a band corresponding to ABCA1 under non-reducing conditions (Figure 6). In the presence of the reducing agent β-mercaptoethanol, free apoA-I was observed at its expected molecular weight of 28 kDa. Like apoA-I, when tyrHDL was DSP crosslinked, a prominent anti-apoA-I antibody reactive band colocalized with ABCA1 under non-reducing conditions, and was released in the presence of reducing agent to a ladder of lower molecular weight apoA-I-containing bands. In contrast to control HDL, which showed very minimal binding to ABCA1, these results suggest conformational changes are present in lipid-bound tyrHDL apolipoproteins that allow them to bind to ABCA1 like lipid-free apoA-I.

### tyrHDL undergoes enteropeptidase digestion similar to lipid-free apoA-I

**Figure 7:**
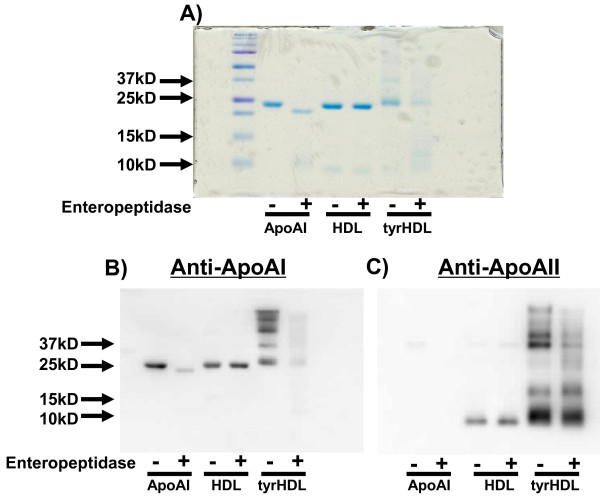
**Enteropeptidase digestion of tyrHDL**. Lipid-free apoA-I, HDL, and tyrHDL were treated with or without enteropeptidase 0.13 U per μg of protein for 6 h at 37°C. Samples were run on 15% SDS-PAGE under reducing conditions and stained with Coomassie Blue (A) or transferred to nitrocellulose for immunoblotting with anti-apoA-I (B) or anti-apoA-II (C). The data is representative of 5 separate experiments with similar results.

To investigate the possibility that lipid-bound apos on tyrHDL can assume a lipid-free conformation, the susceptibility of tyrHDL to enteropeptidase digestion was compared to unmodified HDL and apoA-I. Enteropeptidase cleaves lipid-free apoA-I at Arg^188 ^to form a major fragment of about 22 kDa, but has no ability to cleave lipid bound apoA-I present either on reconstituted or native HDL (15). Enteropeptidase treatment of lipid-free apoA-I converted a majority of the protein to a lower molecular weight fragment (~22 kDa), while HDL was resistant to proteolysis (Figure 7). Exposure of tyrHDL to enteropeptidase resulted in loss of the majority of a 37 kDa band consistent with an apoA-I-A-II monomer heterodimer, both on Coomassie blue-stained SDS PAGE gel and by immunoblotting with anti-apoAI and anti-apoAII. This apoA-I-apoA-II heterodimer has been identified as the protein element of tyrHDL that is responsible for enhanced cholesterol efflux when compared to HDL [9]. Nearly all of the apoA-I in this heterodimer on tyrHDL was digested, whereas the apoAII component was only partially digested, possibly due to apoAII multimers also running at this molecular weight. The apoAI monomer band of tyrHDL was also mainly digested, to fragments smaller than 22 kDa, suggesting apoAI in tyrHDL is susceptible to enteropeptidase but produces different fragments than native apoAI (Figure 7A and 7B). These results suggest that unlike lipid-bound proteins on the surface of native HDL, the lipid-bound apoAI-AII heterodimer, and also tyrosylated apoAI, on the surface of tyrHDL are able to assume at least partial lipid-free conformations, making them susceptible to enteropeptidase digestion.

## Discussion

The current study provides several lines of evidence that oxidation of HDL by peroxidase-generated tyrosyl radical induces conformational changes in lipid-bound HDL apolipoproteins that allows them to interact with ABCA1 like lipid-free apoA-I. Incubation of ABCA1-expressing human fibroblasts with tyrHDL increases total cellular and cell surface ABCA1 and inhibits calpain-dependent and -independent proteolysis of ABCA1, as also mediated by lipid-free apoA-I but not HDL [3,4]. Additionally, tyrHDL can be crosslinked to ABCA1 and is susceptible to proteolysis by enteropeptidase like lipid-free apoA-I. These findings, therefore, represent the first demonstration of apoproteins on the surface of spherical HDL particles behaving like lipid-free apoAI and capable of interacting with and stabilizing ABCA1 against proteolysis although lipid-bound. The tyrHDL-induced increase in ABCA1 protein is consistent with our previously observed ability of tyrHDL to markedly deplete ACAT-accessible cholesterol [6], increase cholesterol available for removal by apoA-I [7], and to reduce atherosclerosis development in apoE-deficient mice [8] in comparison to native HDL.

### Apoproteins on the surface of spherical tyrHDL increase ABCA1 protein level and activity

Our findings indicate that tyrHDL increases cholesterol mobilization from cells by interacting directly with ABCA1 and enhancing ABCA1 protein stability. Like lipid-free apoA-I, no effect was seen by tyrHDL on ABCA1 mRNA levels. Consistent with this, previous studies demonstrated the lipid extract of tyrHDL had no ability to enhance cholesterol mobilization from cells when reconstituted with control HDL apoproteins, suggesting oxysterols present in tyrHDL lipids were not driving ABCA1 expression at a transcriptional level [9]. Those studies also found the lipid-free protein fraction of tyrHDL had a similar but slightly *lower *ability to deplete ACAT-accessible cholesterol than equivalent concentrations of lipid-free proteins from native HDL [9]. tyrHDL proteins reconstituted into discoidal HDL showed a modestly increased ability to deplete this cholesterol pool in comparison to discoidal rHDL made with native HDL proteins. Only when tyrHDL proteins were reconstituted onto the surface of spherical HDL was the full differential activity of tyrHDL compared to native HDL observed [9]. These results suggest the enhanced activity of tyrHDL versus native HDL is most clearly seen when tyrHDL proteins are bound to a spherical rather than a discoidal HDL surface. They also suggest that it is the particular conformation of tyrHDL apoproteins when present on the surface of spherical HDL, and not tyrHDL apos that dissociate off the particle surface, that are responsible for this effect.

### Apoproteins on the surface of tyrHDL appear to assume a partially lipid-free conformation

The combined results suggest HDL apoproteins oxidized by tyrosyl radical and specifically the apoAI-AII heterodimer created by this oxidation binds to the surface of the lipoprotein differently than apoproteins on HDL, and that the modifications induced by tyrosyl radical oxidation produce a partially lipid-free conformation in lipid-bound apoA-I. We propose a possible model wherein the more lipophilic apoA-II component of the apoAI-AII heterodimers on tyrHDL [9] tethers apoA-I to the HDL particle surface, allowing apoA-I to be attached to HDL while it also assumes a partially lipid-free conformation. This conformation would facilitate binding of the tethered apoA1 to ABCA1, and inhibition of ABCA1 degradation. As a result, exposure of cells to tyrHDL causes ABCA1 to be retained on the cell surface and protected from calpain-dependent (cell surface) as well as calpain-independent (intracellular) proteolysis. The increased ABCA1 protein level leads to the enhanced mobilization of excess intracellular cholesterol that would otherwise be esterified, and makes this cholesterol available for efflux to apoA-I and new HDL particle generation.

The nature of the conformation of tyrHDL apoAI-AII heterodimers on the surface of tyrHDL requires further investigation. Particular regions of apoA-I thought to be critical to the apoA-I-ABCA1 interaction have been suggested but not yet confirmed [21-23]. The similarities in binding to and stabilization of ABCA1 between lipid-bound tyrHDL apos and lipid-free apoA-I suggests tyrHDL represents a useful tool to investigate the nature of this apoprotein-ABCA1 interaction critical to the formation of new HDL particles.

Whether or not peroxidase-generated tyrosyl radical oxidation of HDL represents a significant mechanism of HDL oxidation in vivo is an unanswered question, and is not the purpose of these studies. Myeloperoxidase is known to generate tyrosyl radical as one of its oxidizing products in addition to hypochlorous acid [24], and LDL containing products of tyrosyl radical oxidation have been isolated from human atherosclerotic plaques [25]. The relative importance of tyrosyl radical oxidation of HDL in vivo, which ex vivo appears to enhance ABCA1 activity, as compared to other oxidative modifications of HDL and apoA-I that impair ABCA1-dependent cholesterol efflux [26,27], remains to be determined. Regardless, the ability of tyrHDL generated ex vivo to enhance ABCA1 activity represents an important model for the development of peptide therapies that retain this ability when in the lipid-bound form. Since nearly all HDL apoproteins are lipid-bound in vivo, this signifies a major difference from apoA-I, which only shows ABCA1-enhancing activity when lipid-free.

## Conclusions

The results presented here provide the first demonstration of lipid-bound HDL apolipoproteins capable of enhancing ABCA1 protein level and activity like lipid-free apoA-I, and an explanation for the previously identified salutary effects of tyrHDL.

## List of abbreviations

The abbreviations used are: HDL: high density lipoproteins; apo: apolipoprotein; ABCA1: ATP-binding cassette transporter A1; tyrHDL: tyrosyl radical-oxidized HDL; BSA: fatty acid-free bovine serum albumin; DMEM: Dulbecco's modified Eagle's medium; ALLN: N-acetyl-Leu-Leu-norleucinal; ACAT: acyl-CoA:cholesterol acyltransferase.

## Authors' contributions

MAH participated in the design of experiments and writing of the manuscript, and performed the majority of the experiments. SN performed the initial experiments suggesting an interaction of tyrHDL with ABCA1. TC performed experiments for the study and assisted with figure generation and writing of the manuscript. MNO provided intellectual input and assistance with the writing of the manuscript. GAF designed the experiments, oversaw the project and data analysis, and wrote most of the manuscript. All authors read and approved the final manuscript.
